# Progress in Medicine

**Published:** 1899-07-29

**Authors:** 


### Progress in Medicine.

### NEUROLOGY.

*(Continued from page 278.)*

The prognosis in cerebral hæmorrhage is discussed as follows by Browning.[1] There are two separate questions to be considered, the continuation of life and the extent of recovery from the attack. In the rare attacks of childhood, improvement may be expected for some years though entire recovery is unusual. In middle life where the motor involvement is not great, practically complete restitution of all functions is occasionally observed. More often some impairment persists. In senile conditions but limited recovery is to be expected. The subsequent length of life depends much on the care and kindness with which the chronic invalid is surrounded. The great guide to prognosis is the severity and nature of the attack. Coma, stertor, vomiting, prolonged semi-consciousness, extensive and complete paralysis indicate a large effusion. Consequently there is immediate danger to life. Prolonged high temperature makes a fatal prognosis probable. General convulsions, as indicative of ventricular rupture, are of particularly bad omen. Supposing the acute stage has been tided over, if the tendon jerks are increased on the paralysed side, some permanent injury will remain. The occurrence of œdema or contractures in the paralysed part precludes hope of recovery. The anæsthesias that are so frequently present in the early stage rarely prove lasting, and the occasional development of chorea in the affected extremities is so far a good sign, as it indicates returning conductivity of the motor tracts.

**Epilepsy.**—Bechet[2] has made a careful study of forty families, epileptic members of which had come under observation, to elucidate the hereditary antecedents and mode of transmission of the disease. He found that the duration of life is sensibly less among the antecedents of epileptics than that which normal families present.

Epileptics belong generally to large families, but they are not themselves able to beget large families—they tend to sterility. Epileptic families have a lower vitality than normal families. The morbidity in the ancestry and issue of epileptics takes a special form, pulmonary diseases and especially phthisis being common in the parentage, whilst cerebral affections and specially meningitis are commonly found among the children of epileptics; insanity proper is rare in epileptic families.

**Bromide of Strontium.**—This is highly recommended in place of the potassium salt by Roche,[3] who gives one drachm a day as a rule in infusion of quassia. Should the fits recur with this dose, it is increased to as much as three drachms. The bromine rash is sometimes troublesome, but can be controlled by a few minims of liquor arsenicalis.

Suckling[4] gives the following prescription in epilepsy: ℞. Pot. bromid gr. x., ammon. bromid gr. x., tinct. bellad. m. x., aq. menth. pip. ℥i. Two tablespoonfuls to be taken on getting into bed, and one tablespoonful on rising in the morning; this mixture may be increased or diminished if necessary. If the medicine is ordered in this way the patient does not feel the worry of taking it, as he would if he had to take it in the daytime. If the bromide mixture fails borax should be tried, or ergot. Bromide of strontium is not so useful as the potassium or ammonium salts. Arsenic is of no use, and if given additionally for acne may bring on an attack. In epilepsy with a slow pulse (40 to 50), bromides, combined with trinitrin may stop the attack. Where the bromides have failed zinc salts are useless. The best method of treatment of status epilepticus is to clear the bowels out with a large enema and then to inject 1-100th of a grain of hydrobromide of hyoscine. More than two injections are rarely needed.

**Paralysis Agitans.**—Williamson[5] has recently discussed the palliative treatment of this disease, the progress of which cannot be checked by any medicinal treatment. It is of the greatest importance to secure sound sleep, as a restless night is quite sufficient to make the symptoms worse on the following day. Sulphonal, or whisky and hot water are often effectual in producing sleep, though alcohol taken during the daytime seems to increase the trembling. Tea and coffee often have the same effect. Needlework or writing often seem to diminish the trembling. Systematic open air treatment, carriage drives, for instance, is often beneficial. Strychnine, arsenic, potassium iodide, calabar bean, gelseminum, cannabin tannate, are all of no good. Morphia, hypodermically, certainly gives relief, but is objectionable in a disease of such a chronic course. Morphia, or duboisin by the mouth, has little effect. Hyoscine is the only drug which is really useful. The dose is 1-96th gr. of the hydrobromate dissolved in chloroform water, which may be increased to 1-75th gr. In these doses hyoscine diminishes the tremblings and the restlessness of the muscles and the uneasiness, and no bad effects are observed. Though this dose by the mouth could be easily borne, yet 1-75th gr. hypodermically might produce alarming results. After some weeks the drug loses its good effect, when it is best to stop it for a time; on recommencing it the good effect is again obtained.

**Chorea.**—Lichtschein[6] records three cases of severe chorea treated by prolonged chloral sleep, with excellent results. The treatment is especially applicable to those cases in which there is no fever and no heart disease, but where the movements are severe and continuous. The chloral has, in all probability, no curative influence of itself over the chorea, except through the intervention of the sleep which it induces. No more should be given than is absolutely needed to maintain the continuous sleep. The period in which the patient is awake should, as far as possible, not exceed half-an-hour. Fluid nourishment should be given five or six times a day, and the use of the bedpan should be had recourse to. From 20 to 30 grains every two or three hours is necessary at first to keep the patient in a deeply somnolent condition; after a time this can be reduced to 15 or 10 grains. If there is any cardiac weakness 1-200th gr. of sulphate of strychnine can be added to each dose of the chloral. As a rule, two or three weeks' treatment is sufficient to effect a complete cure.

**Retrobulbar neuritis.**—Sym,[7] in an article on this affection, says the term was first introduced by Von Graefe to indicate optic neuritis arising primarily in the nerve or by continuity with other parts, and not from intracranial mischief. Rapid failure of vision, usually in one eye only, often accompanied by pain and tenderness in the neighbourhood, absence of early ophthalmoscopic changes, and a tendency to recover are the usual prominent features. The failure of vision is of a peculiar type; it is principally or solely central; its degree varies from a loss of definition to complete loss. In the less severe cases there is central loss of colour perception, red and green appearing as yellow and white respectively. Many patients say that vision improves in the evening; vision is better after a night's rest. The patient is apt to complain of a mist or fog before the eye. Temporary attacks of blindness—often found in ordinary neuritis—are never present. Apart from toxic amblyopias, retrobulbar neuritis not infrequently affects both eyes consecutively. Ophthalmoscopic signs are usually absent early, and may be, though not usually, altogether absent. But they are trifling in proportion to the loss of vision—in contrast to ordinary neuritis, where the ophthalmoscopic signs may be great, and visual symptoms absent. The prognosis usually is good. Improvement often begins in a few weeks or days, sometimes coinciding with the appearance of ophthalmoscopic signs of neuritis. In a large proportion recovery is complete; in some a scotoma persists. The unilateral cases may be due to spread to the nerve of local inflammation, or to syphilis, gout, rheumatism, or malaria. They do not differ from the bilateral except in the tendency to recovery. The latter occur in diseases of the spinal cord, especially tabes, disseminated sclerosis, and general paralysis; they include hereditary optic atrophy and toxic amblyopias. The treatment of the unilateral cases consists in attacking vigorously the constitutional cause. Action of the skin and kidneys must be encouraged. Pilocarpine subcutaneously will be found as useful as in persistent scleritis. Some believe in iodide and mercury even in non-syphilitic cases. In longer standing cases strychnine is chiefly to be relied on.

Schweinitz[8] records two cases of retrobulbar neuritis with facial palsy. The first patient had, first, right facial palsy; two years later right retrobulbar neuritis, followed one and a half months later by a mild left retrobulbar neuritis; ultimate complete recovery. The second patient had two attacks of right facial palsy, followed two years later by a severe retrobulbar neuritis. It would seem, then, that the same cause operative in the same susceptible subject may produce either neuritis in the Fallopian canal, or neuritis in the optic foramen.

Flemming[9] also records a case of retro-ocular neuritis of the left eye in a woman aged 22 years, probably due to spreading of inflammation from the nasal cavity. Vision was less than 6-60ths. The eyeball was distinctly tender when pressed back into the orbit; the media were clear; the optic disk was a little too red, and the margins slightly blurred. There was a marked central scotoma in the left field of vision. The special point to which attention was directed was that the left pupil reacted to light, but the contraction was only momentarily maintained; on the other hand, it reacted well to indirect stimulation—the light falling on the right eye —and this contraction was maintained. This showed that whilst the efferent path for the light reflex in the left eye was intact the afferent path was damaged.

**Acute Alcoholism.**—Dana[10] has studied the pathological changes found in the brain of cases dying from acute alcoholism. He finds that patients who died of alcoholism with all the symptoms of meningitis showed simple congestion of the pia-arachnoid with some œdema of its texture. Microscopical examination rarely showed any migration of leucocytes or anything approaching encephalitis. The larger (pyramidal and giant) nerve cells showed pigmentation to an intense degree, the pigment being diffused through the cell body; and the cytoplasm showed various degrees of fatty and granular degeneration. These appearances indicate serious nutritive changes in the cerebral cells from the direct toxic effect of the alcohol.

Ewing[11] has also investigated the changes in nerve cells due to acute alcoholism. The motor cells of the cord and medulla were extensively affected. Chromatolysis was evident, and the cell body showed hyaline degeneration. The outline of many cells were irregular and ragged, and the nuclei were dislocated from their normal position. Similar changes were found in the cortical cells of the hemispheres. The nervous symptoms of delirium tremens are ascribed in large measure to the profound nutritive changes, as revealed above, which alcohol produces, and which give rise to restlessness, tremor, insomnia, and pyrexia before they end in a fatal issue.

**Facial Paralysis of Aural Origin.**—Mounier[12] says that in all cases of facial paralysis the ear should be examined. In acute otitis media without perforation of the drum a collection of fluid in the tympanum may compress the facial nerve in the Fallopian canal which often opens into the middle ear. The accompanying pain is often attributed to the nerve disease, and in the absence of discharge otitis is not thought of. Opening of the tympanum is the only mode of treatment. Sometimes spontaneous perforation of the drum is insufficient to remove the pressure on the facial nerve. Chronic suppurative otitis with caries is the most common cause of facial paralysis. Formerly such cases were thought to be beyond the resources of surgery, but even in the most chronic compression may be the cause of the paralysis, and something may be done by thoroughly clearing out the middle ear and antrum.

**Peripheral Neuritis.**—Bury[13] records a case of multiple peripheral neuritis characterised by extensive motor paralysis, with marked vaso-motor disturbance, ending in symmetrical gangrene of the feet. The post-mortem examination showed that the spinal cord was intact, and that there was extensive degeneration of the intra-muscular portions of the nerves. The occurrence of gangrene indicates that occasionally vaso-motor nerves may be picked out by neuritis, the symptoms resulting therefrom being identical with those of Raynaud's disease, though the symptoms of Raynaud's disease do not always depend on lesions of vaso-motor nerves, but may in some cases be related to a morbid condition of the vaso-motor centres. The term Raynaud's disease, like Landry's paralysis, probably includes different groups of case, which, although presenting similar or even identical clinical features, are dissimilar as regards the location of morbid action.

**Neurotic Ulcers of the Mouth.**—Sibley[14] records three cases of this rare disease, which has usually been described as of the nature of pemphigus, though its distribution is sometimes suggestive of herpes. It, however, differs from pemphigus in its persistence through many years. It generally begins as a crack or streak, or from the beginning as a small superficial bright red ulcer. There is a good deal of burning sensation and great distress, accompanied by profuse salivation, and if the ulcer is very indolent there is œdema of the parts around. Mental trouble appears to be the cause of the ulcers, which are probably produced by a distinct tropho-neurosis, and are quite different from the ordinary catarrhal or dyspeptic ulcer. No medicinal treatment appears to give any very obvious result; possibly some nerve tonics may do good indirectly, and local applications may relieve the distress and pain for the time, and assist in the more rapid healing of the sores. Many cases accompanied by severe pain are relieved by cocaine and borax, carbolic, or peroxide of hydrogen mouth washes. Opium in moderate doses at night does good. The only curative treatment is complete removal from home and its accompanying worries, and it appears that mountain air is preferable, whilst sea voyages have sometimes made matters worse.

[1] Browning, Clin. Jour., May 3, 1899. [2] Bechet. [3] Roche, Lancet, April 22, 1899. [4] Suckling, Birm. Med. Rev., Mar., 1899. [5] Williamson, Brit. Med. Jour., June 3, 1899. [6] Lichtschein, Med. Rec., April 1, 1899. [7] Sym, Amer. Jour. of Med. Sci., Feb., 1899. [8] Schweinitz, Jour. of Nerv. and Med. Dis., May, 1899. [9] Flemming, Practitioner, May, 1899. [10] Dana, Quar. Jour. of Inebriety, Jan., 1899. [11] Ewing, Archiv. of Neur. and Psycho-path., Vol. I. [12] Mounier, La France Médicale, Feb. 12, 1899. [13] Bury, Med. Chron., April, 1899. [14] Sibley, Brit. Med. Jour., April 1, 1899.

